# Evolutionary conservation of domain-domain interactions

**DOI:** 10.1186/gb-2006-7-12-r125

**Published:** 2006-12-21

**Authors:** Zohar Itzhaki, Eyal Akiva, Yael Altuvia, Hanah Margalit

**Affiliations:** 1 Department of Molecular Genetics and Biotechnology, Faculty of Medicine, The Hebrew University of Jerusalem, Jerusalem 91120, Israel

## Abstract

**Background:**

Recently, there has been much interest in relating domain-domain interactions (DDIs) to protein-protein interactions (PPIs) and *vice versa*, in an attempt to understand the molecular basis of PPIs.

**Results:**

Here we map structurally derived DDIs onto the cellular PPI networks of different organisms and demonstrate that there is a catalog of domain pairs that is used to mediate various interactions in the cell. We show that these DDIs occur frequently in protein complexes and that homotypic interactions (of a domain with itself) are abundant. A comparison of the repertoires of DDIs in the networks of *Escherichia coli*, *Saccharomyces cerevisiae*, *Caenorhabditis elegans*, *Drosophila melanogaster*, and *Homo sapiens *shows that many DDIs are evolutionarily conserved.

**Conclusion:**

Our results indicate that different organisms use the same 'building blocks' for PPIs, suggesting that the functionality of many domain pairs in mediating protein interactions is maintained in evolution.

## Abstract 2

Mapping of domain-domain interactions onto the cellular protein-protein interaction networks of different organisms demonstrates that there is a catalogue of domain pairs that is used for mediating various interactions in the cell

## Background

Many proteins are constructed of domains, which are their main functional and structural units. A specific domain can be found in different proteins, and several different domains can be found within a given protein. Proteins can thus be viewed as being built of a finite set of domains, which are joined together in diverse combinations. Domains are often related to particular functions; for example, they may be responsible for catalytic activity or they may mediate the interactions of proteins with other molecules [1-3]. They are believed to play a crucial role in protein-protein interactions (PPIs), by binding either short peptide motifs or other domains. The former are usually associated with transient interactions, whereas the latter are assumed to mediate more stable interactions and assemblies of proteins into complexes [2]. Domain-domain interactions (DDIs) can be either heterotypic, when the interaction involves two different domains, or homotypic, when it involves two identical domains. Homotypic interactions do not necessarily imply the formation of homodimers but may also involve binding of two different proteins or intraprotein interactions mediated by two identical domains. Heterotypic interactions refer to interactions between two different domains either within a protein or between proteins (different or identical).

The domain modularity of proteins on the one hand and the fact that PPIs are mediated via DDIs on the other hand raise the question of PPI modularity; can the PPIs be attributed to a limited set of DDIs? Two lines of evidence support this idea. The first comes from the work of several groups who found statistically significant over-representation of domain pairs in large datasets of experimentally determined PPIs [4-11]. The inferred domain pairs can be considered as putative interacting domain pairs that are shared by multiple PPIs. In some cases these putative DDIs could indeed be supported by available experimental data (for example, see the report by Sprinzak and Margalit [4]) and/or confirmed by structural information from solved protein complexes (for example, see the report by Riley and coworkers [11]). However, in most cases experimental verification in support of the DDI-PPI correspondence is still missing. The second line of evidence comes from structurally based DDI databases that were recently published [12,13] and list the actual domains that are involved in the interactions, based on solved structures from the Protein Data Bank [14]. These databases include many DDIs that are shared between different PPIs, corroborating the modularity of PPIs. However, because the dataset of crystallograpically solved PPIs is relatively small, it is not clear whether we can conjecture from it to the cellular PPI networks.

In the present study we combined the structurally derived information with the PPI network information based on small-scale and large-scale experiments, in order to study further the modularity of the PPIs. It is well known that domains often exhibit evolutionary conservation in sequence and three-dimensional structure [15], and therefore it might be expected that the same domain pairs mediate PPIs in different organisms. It is intriguing, therefore, to examine whether there are common DDIs that can be identified in the PPI networks of the various organisms. To this end we mapped the structurally determined DDIs onto the PPI networks of five organisms (*Escherichia coli*, *Saccharomyces cerevisiae*, *Caenorhabditis elegans*, *Drosophila melanogaster*, and *Homo sapiens*) and compared the occurrence of these interacting domain pairs in the studied networks to that expected at random. This analysis provides a proteome-wide view on the involvement of these interacting domain pairs in protein interactions in the cell. Next, we compared the DDI repertoires of the five organisms and showed that there are DDIs that are unique to a specific organism; DDIs that are shared by two, three, or four organisms; and DDIs that are conserved in all five organisms. Many of the highly conserved DDIs involve domains known to function in basic processes, such as DNA metabolism and nucleotide binding. In summary, our results suggest that different organisms use the same 'building blocks' for PPIs and that the functionality of many domain pairs as mediating protein interactions is maintained in evolution.

## Results

### Database of DDIs

Recently, two databases of DDIs based on high-resolution three-dimensional structures were published, namely the database of 3D Interacting Domains (3DID) [12] and the iPfam database [13], both derived from the Protein Data Bank [14]. These databases contain information from a variety of organisms, ranging from bacteria to human. The DDIs in these databases are based on two types of interactions: interprotein DDIs (interactions between domains in two different proteins) and intraprotein DDIs (interacting domains within multidomain proteins). The 3DID and iPfam databases differ slightly in their DDI definitions and therefore they overlap in only about 70% of the DDIs. We combined the DDI data from both databases and filtered it as described in the Materials and methods section (below), resulting in a database that contained 2,983 DDIs. Of these DDIs, 74% were derived from interprotein interactions, 13% were derived from intraprotein interactions, and 13% DDIs were found in both interprotein and intraprotein interactions (Additional data file 1 [Supplementary Figure 1a]). Some DDIs occurred only once whereas others appeared repeatedly (up to hundreds of times). The median number of DDI occurrences was nine. This already suggests that there are domain pairs that are used repeatedly in different interactions.

### DDIs as the building blocks of cellular PPI networks

**Figure 1:**
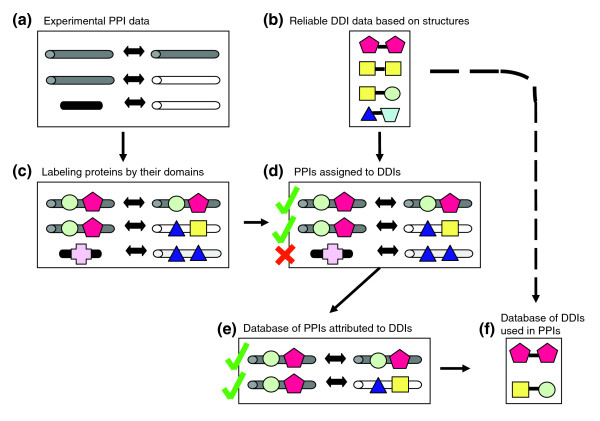
A schematic description of the analysis. **(a) **A list of experimentally determined PPIs is compiled for each of the five organisms (*E. coli*, *S. cerevisiae*, *C. elegans*, *D. melanogaster*, and *H. sapiens*) from INTACT [32], DIP [19], and BIOGRID [33]. **(b) **A list of structurally derived DDIs is compiled from 3DID [12] and iPfam [13] databases. **(c) **The appropriate domains are assigned to each of the interacting proteins according to the definitions of the InterPro database [34]. **(d) **Based on the data complied in panels b and c, DDIs are mapped onto PPIs. **(e) **A list of PPIs with DDI assignments is compiled. **(f) **A list of the DDIs mapped onto PPIs is compiled. DDI, domain-domain interaction; PPI, protein-protein interaction.

Next, we asked whether the DDIs can be identified in the cellular PPI networks of various organisms (*E. coli*, *S. cerevisiae*, *C. elegans*, *D. melanogaster*, and *H. sapiens*) in a frequency that exceeds random expectations. We mapped the DDIs onto the PPI networks, as described in Figure 1. This mapping allowed us to focus on the PPIs that may be mediated by the DDIs in each of the organisms (Figure 1e) and to study the repertoire of DDIs in each organism (Figure 1f). Interestingly, DDIs derived solely from intraprotein interactions could be mapped onto only a very small fraction of PPIs. Most PPI-DDI mappings involved DDIs from interprotein interactions, and some mappings involved DDIs derived from both interprotein and intraprotein interactions (Additional data file 1 [Supplementary Figure 1b]).

The fractions of the organisms' PPIs with domain assignments to which DDIs could be mapped ranged from 6% to 20% (Table 1). To evaluate whether the number of interactions attributed to DDIs is statistically significantly greater than expected at random, we generated 1000 same size, same topology, organism-specific random PPI networks (see Materials and methods). For each of these networks we counted the number of PPIs to which structurally based DDIs could be mapped. The fraction of random networks in which the number of interactions attributed to DDIs was equal to or exceeded the number in the studied network provided a measure of statistical significance. Our analysis revealed that for each of the five organisms the number of PPIs attributed to DDIs was statistically significantly greater than expected at random (Table 1).

Both the 3DID and iPfam databases are based on a variety of organisms, and there is some overlap between the PPIs in the organisms' networks and those used to derive the structurally based DDIs. To rule out a potential bias in the results due to this overlap, we repeated the analysis for each organism disregarding PPI-DDI mappings caused by overlap between the structural database and the PPI data of that organism. As expected, there was a slight decrease in the number of PPIs attributed to DDIs in the various organisms, but these numbers remained highly statistically significant (Table 1).

Our statistical evaluation strongly supports the conjecture that PPIs in the cellular networks may use the structurally based interacting domain pairs to mediate their interactions. Still, without explicit structural information, there is always the possibility that in multidomain proteins the mapped DDIs are not actually the domains that mediate the interaction for particular interacting protein pairs. We therefore turned to examine a subset of the PPIs, namely those involving only single domain proteins. We first verified that the domains constitute most of the sequences of these single domain proteins, and therefore it is conceivable that these PPIs are mediated by residues within the domains. As shown in Table 2, the number of single domain PPIs that could be attributed to the DDIs highly exceeded random expectation. This further supports our previous conclusion that there are domain pairs that are used preferentially for PPIs.

**Figure 2:**
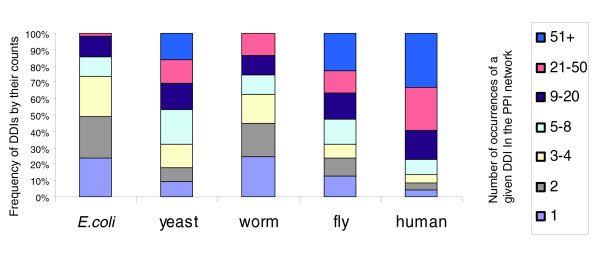
Repeated use of interacting domain-pairs in PPI networks. For each organism, the number of occurrences of each DDI in the PPI network was counted. The histogram shows the frequency of PPIs that were attributed to DDIs used only once, twice, and so on. The frequency is computed out of all the PPI-DDI mappings. DDI, domain-domain interaction; PPI, protein-protein interaction.

**Figure 3:**
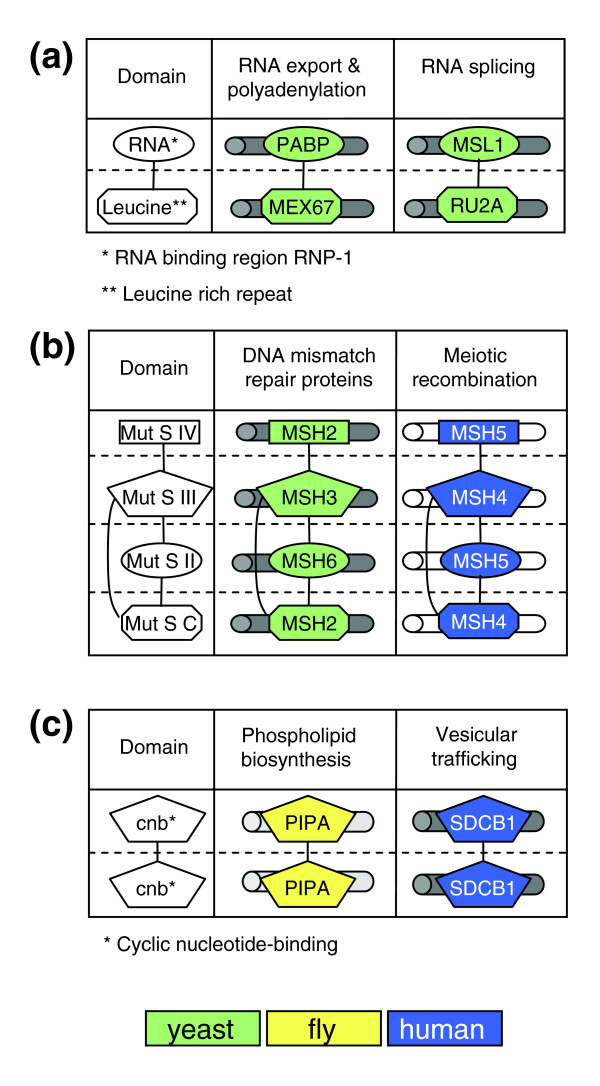
The same DDIs are used in different cellular contexts and in different organisms. The interacting domains (demonstrated and labeled in the left-most column) were mapped onto the interacting proteins (demonstrated and labeled in the two right columns). Edges connect between interacting domains/proteins. The proteins may be multidomain proteins, but only the relevant domain is demonstrated. **(a) **An example of the same DDI mapped onto two pairs of interacting proteins in yeast, which are involved in different processes, namely RNA export and RNA splicing. **(b) **An example of a subnetwork of four proteins whose interactions are attributed to the same DDIs in *S. cerevisiae *and in human. In yeast, the interacting proteins are involved in DNA mismatch repair and in human they are involved in meiotic recombination. The proteins MSH4 and MSH5 are not considered homologs of the proteins MSH2, MSH3, or MSH6 (based on the report by Altschul and coworkers [40] and on sequence comparison). **(c) **An example of two PPIs attributed to the same DDI in different processes in *D. melanogaster *and human; in fly it is involved in phospholipid biosynthesis and in human in vesicular trafficking. These examples emphasize the modularity of DDIs and their possible role as the 'building blocks' of the PPI networks. The Swiss-Prot accessions of the proteins are as follows: PABP: [P04147]; MEX67: [Q99257]; MSL1: [P40567]; RU2A: [Q08963]; MSH2: [P25847]; MSH3: [P25336]; MSH6: [Q03834]; MSH5: [O43196]; MSH4: [O15457]; SDCB1: [O00560]; and PIPA: [P13217]. DDI, domain-domain interaction; PPI, protein-protein interaction;. fly, *D. melanogaster*; yeast, *S. cerevisiae*.

Our analyses defined for each organism a set of interacting domain pairs that can be considered as mediating the PPIs, as illustrated in Figure 1f (and detailed in Additional data file 2). The counts of DDIs that were mapped onto the PPI network of each organism are summarized in Table 3. These counts greatly exceeded the corresponding numbers in random networks (*P *< 0.001). Figure 2 describes the distribution of these DDIs among the organisms' PPIs. Although in each organism there are DDIs that are mapped only to one PPI, most DDIs are mapped to two or more PPIs. Notably in human, at least 20% of the PPI-DDI mappings were attributed to a relatively small number of DDIs. Each of these DDIs was mapped to more than 90 PPIs. Because there is always the concern that certain DDIs are over-represented due to paralogs that carry out paralogous interactions, we also carried out the analysis after excluding paralogous interactions. The exclusion of paralogous interactions resulted in a significant decrease in the number of repeatedly used DDIs in *E. coli *to 81 DDIs (approximately 10% of *E. coli *DDIs), but had a much smaller effect on the other organisms (Additional data file 1 [Supplementary Table 1]). For the eukaryotes, the fractions of PPI-DDI mappings attributed to repeatedly used DDIs were still very high, and ranged between about 72% to 96% when paralogous PPIs were excluded (Additional data file 1 [Supplementary Figure 2]). These findings support the conjecture of Dueber and coworkers [16] on the higher functional flexibility that proteins in eukaryotes may achieve by using the same domains for interactions in different contexts. This is also exemplified in Figure 3a, in which the use of the same DDI to mediate PPIs in different processes within the same organism is demonstrated. Our findings support previous reports based on *S. cerevisiae *data [4,5], and imply that at the organism level there are pairs of domains that can be considered the 'building blocks' of the PPI networks, and these are used in different protein contexts to mediate the interactions.

### DDIs are evolutionarily conserved

**Figure 4:**
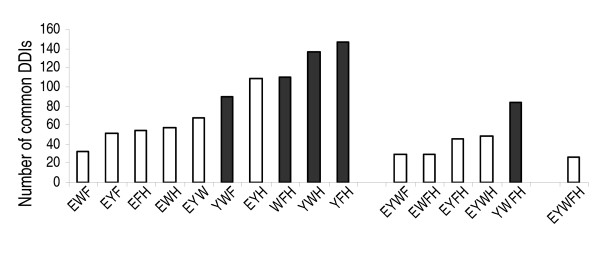
Interacting domain pairs shared by several organisms. The histogram shows the number of DDIs shared by three, four, and all five organisms. White bars represent DDIs that are used also in *E. coli *and black bars represent DDIs common only to the eukaryotes in our study. Twenty-seven DDIs were shared by all five organisms. E: *E. coli*. Y: yeast (*S. cerevisae*). W: worm (*C. elegans*). F: fly (*D. melanogaster*). H: human. DDI, domain-domain interaction.

Are these 'building blocks' conserved in evolution? To address this question we compared the repertoires of DDIs of the different organisms, and examined how many of the DDIs are common to two, three, four, or all of the five organisms. The results are described in Table 3 and Figure 4 (also see Additional data file 1 [Supplementary Table 2]). For each such comparison, the number of common DDIs was compared with their number in the intersection of 1000 random DDI networks of the compared organisms, to obtain a measure of statistical significance (see Materials and methods, below). Because many of the structurally derived DDIs were determined from human and *E. coli*, it is not surprising that the number of unique DDIs is high for these organisms and low for the other organisms (Table 3). It is important to emphasize that most of the organisms' unique DDIs are not due to organism-specific domains. These domains occur in the proteins of other organisms, but PPIs that contain these DDIs were not determined yet. Table 3 shows the common DDIs between all pairs of organisms. Again, most of the DDIs of yeast, worm, and fly are shared with either *E. coli *or human, because many of the DDIs were taken from structures of these two organisms. However, it is instructive that other organism-organism comparisons revealed high numbers of common DDIs, which were all statistically significant. It is clear from Table 3 and Figure 4 that the similarity in DDI repertoires is much higher among the eukaryotes than between *E. coli *and the eukaryotes. Figure 3 demonstrates two examples of the use of the same DDIs in human and either yeast (Figure 3b) or fly (Figure 3c). As seen in the figure, the same DDIs are used in the various organisms in different cellular processes.

The intersections of three, four, or five DDI sets were even more revealing. As demonstrated in Figure 4, when *E. coli *was included in the comparisons the number of common DDIs was rather small. However, when comparing three or four eukaryotes the number of common DDIs ranged between 84 and 147 (*P *< 0.001). Exclusion of interologs (see Materials and methods, below) hardly affected these numbers (Additional data file 1 [Supplementary Table 2 and Supplementary Figure 3]). Many of these DDIs are homotypic (involving identical domains in the interactions). This is already evident in the structural database of DDIs and is reinforced when one examines the conserved DDIs. On average, the repertoire of DDIs of each organism included 56% homotypic DDIs. This fraction increased to 62%, 70%, 77% and 85% among the DDIs that were conserved in two, three, four and five organisms, respectively. These homotypic DDIs are found in both homodimers and heterodimers.

There are 27 DDIs that were found to be conserved among all five organisms and are thus conserved from prokaryotes to eukaryotes (Additional data file 1 [Supplementary Table 2] and Additional data file 3). A close look at these DDIs reveals that they are involved in basic functions such as ATP and nucleic acid binding. Some of the domains involved in these DDIs were documented originally as either prokaryotic or eukaryotic domains, but they occur also in eukaryotic and prokaryotic organisms, respectively, participating in the same or similar functions. In addition to these 27 DDIs, an additional 57 DDIs were found to be shared by the four eukaryotes in our study, mostly involving domains that are characteristic of nuclear proteins and domains that function in protein modification and signal transduction. Looking at DDIs shared by three eukaryotes shows additional common functions, such as intracellular protein transport, which is common to yeast, worm, and human. Focusing on those DDIs conserved in two eukaryotes reveals advanced processes, such as domains involved in dynamics of cytoskeletal elements that are conserved between fly and human.

### DDIs are over-represented in protein complexes

**Figure 5:**
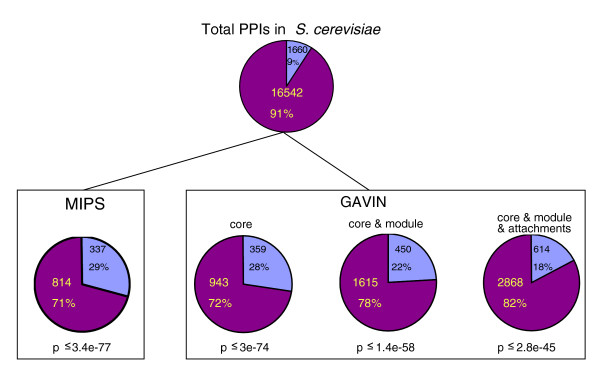
Interacting domain pairs are abundant in protein complexes. The frequency of DDIs in *S. cerevisiae *complexes is statistically significantly higher than their fraction in the whole interactome (*P *values were determined by χ^2 ^test). The fraction of PPIs attributed to DDIs increases with the reliability of the interaction and is highest in the cores of the complexes. DDI, domain-domain interaction; PPI, protein-protein interaction.

It is commonly acknowledged that transient interactions involve short motifs whereas more stable interactions, such as the ones found in protein complexes, are mediated by DDIs [2]. Accordingly, we would expect that the fraction of PPIs attributed to DDIs within stable complexes will be higher than in the whole PPI network. To test this, we examined two datasets that contain information on protein complexes in *S. cerevisiae *[17,18]. The first database, MIPS, is manually curated and is considered to be highly reliable [17]. The second database is based on a highly sensitive large-scale study conducted by Gavin and coworkers [18], in which the proteins involved in complexes were classified into cores, modules, and attachments. Based on the work of Gavin and coworkers, cores contain the most reliable members of the complex, attachments contain less reliable participants, and modules are attachments that recur in several complexes. We marked the PPIs that reside in these complexes, and examined the DDI-PPI mapping for these interactions. Figure 5 shows the fractions of PPIs onto which DDIs could be mapped in the various datasets of complexes, in comparison with the whole yeast interactome. It is clearly seen that in every dataset there is enrichment in PPIs attributed to DDIs compared with their fraction in the entire PPI network (*P *values range between 3.4 × e^-77 ^to 2.8 × e^-45 ^by χ^2 ^test). It is remarkable that the MIPS data and the core data reported by Gavin and coworkers exhibited the greatest enrichment, and as we added more remote components of the complexes (modules and attachments) the fractions of PPIs attributed to DDIs decreased. This supports the involvement of DDIs in more stable interactions.

## Discussion

Using a compilation of structurally derived interacting domain pairs [12,13], we show that experimentally determined interacting protein pairs are statistically significantly enriched in these domain pairs. This suggests that there is a limited catalog of domain pairs that is used to mediate various interactions in the cell and that this catalog is shared to various degrees by different organisms. The fact that the domain regions cover large fractions of the sequences in our study (see Materials and methods, below) further strengthens this finding. Nevertheless, it should be noted that our conclusions are based on putative mappings of the DDIs onto the PPI networks. Until these complexes are solved crystallographically, there is no certainty that these are indeed the domains that mediate the interactions. However, several considerations corroborate our conclusions. First, in the structural databases we also find repeated use of the same DDIs in different PPIs or complexes and in different organisms. Second, our results are highly statistically significant; the counts of PPIs attributed to DDIs in 1000 random networks are always substantially smaller than their counts in the actual networks. Third, we show that PPIs that involve only single-domain proteins are also statistically significantly enriched in the structurally derived DDIs. Fourth, we find the structurally derived DDIs in the PPI networks of various organisms and show that their conservation is statistically significant. Finally, we show that protein complexes that are believed to involve DDIs are enriched in the structurally derived DDIs. All of these findings support the identified DDI-PPI correspondence. Previous studies carried out statistical analyses of all possible domain combinations in a dataset of PPIs and identified over-represented pairs that were suggested as the domain pairs responsible for the interaction [4,7,9]. Demonstrating this phenomenon based on structurally derived DDIs further supports this conjecture, and is important both for understanding the molecular basis of the interactions and as a basis for the identification of new interactions.

Although our results are highly statistically significant, the fractions of PPIs with annotated domains that have been attributed to the structurally derived DDIs are relatively small, ranging from 6% in *D. melanogaster *to 20% in human. This may be due to the lack of information for many DDIs that probably play a role in mediating the interactions, but have not yet been found in solved structures and therefore were not included in this analysis. It is conceivable that with the increase in the number of solved structures, the number of DDIs will increase, followed by an increase in the PPIs that can be attributed to DDIs. A first clue in this direction can be obtained by the expansion of the catalog of DDIs by additional DDIs derived from interactions between single-domain proteins. For single-domain interacting proteins there is almost no doubt as to the domains that may be involved in the interactions, and therefore they provide information that is almost equivalent to domain-level information from solved structures. In our data, some of the single-domain PPIs could be attributed to the structurally derived DDIs, but there were many others that were not classified by the DDIs (Table 2, row iii). These can be used to expand the dataset of DDIs. Indeed, inclusion of the single-domain interacting pairs extended the database of DDIs from 2,983 to 8,228 DDIs. Repeating the same analysis, using this extended DDI database, resulted in the mapping of 20% to 32% of the known PPIs with annotated domains by these defined DDIs (Table 4). These highly statistically significant results (*P *< 0.001) add further support to the suggestion that there is a finite set of interacting domain pairs mediating the PPIs, and that these domain pairs could be considered as the 'building blocks' of the interaction networks.

Assuming that the DDIs mediate more stable interactions, can we evaluate the fractions of stable and transient interactions in the interaction networks from the fractions of PPIs attributed to DDIs? Clearly, our analyses provide an overestimate of the transient interactions and an underestimate of the stable interactions, because not all proteins could be annotated by their domains and because there are probably more DDIs but they have not yet been identified in solved structures. In addition, we used in the analysis interactions based on both large-scale and small-scale experiments. It is possible that a fraction of the interactions that were not attributed to the DDIs do not necessarily represent transient interactions but include false-positive interactions based on large-scale experiments. Indeed, when we repeated the analysis for yeast, including only interactions based on small-scale experiments as reported in the DIP database [19], we obtained a fraction of 20% PPIs attributed to DDIs (versus 9% for PPIs based on both small-scale and large-scale experiments). Thus, in this regard our analysis can be considered as providing a rough estimate of the minimal fraction of stable PPIs in the cellular networks.

Of the DDIs in the structural databases, 399 (13%) were derived from both DDIs within a protein ('intraprotein interactions') and interactions between proteins ('interprotein interactions'). This finding has two implications. First, it provides reassurance for use of DDIs derived from either interprotein or intraprotein interactions for analyzing the domain pairs in PPIs. Second, it lends structural support to the inference of PPIs or functional relationships in cases where domains A and B are found in two different proteins in one organism whereas they are fused into a single protein in a different organism, as suggested by Marcotte and coworkers [20] and Enright and colleagues [21]. The mapping of DDIs onto PPIs further strengthens this conjecture, as 23% to 32% of the PPIs attributed to DDIs in the aforementioned organisms are based on this subset of DDIs (Additional data file 1 [Supplementary Figure 1b]). As already pointed out by Tsoka and Ouzounis [22], we find that many of these DDIs are involved in metabolic processes. Interestingly, although in the structural databases and in our mapping, many of the DDIs are homotypic; this phenomenon is not observed here. Of the DDIs derived from both intraprotein and interprotein interactions, 62.4% are heterotypic interactions and only 37.6% are homotypic interactions. This is in accord with the recent report by Wright and coworkers [23], who suggested that homologous domains in proteins accumulate mutations in order to avoid aggregation. Avoidance of homotypic interactions within proteins should have even a stronger influence toward preventing aggregation. Surprisingly, we found that a very small fraction of the PPIs (2% to 4%) were attributed to DDIs derived solely from intraprotein interactions. The analysis of Littler and coworkers [24] may provide an explanation for this finding. This study pointed out that most of the adjacent domains within a single polypeptide chain (separated by a short loop) tend to interact with one another. This may suggest that some of the intraprotein DDIs may occur merely because of the vicinity of the two domains in the sequence, and would not necessarily occur between two proteins.

Our finding that homotypic DDIs constitute, on average, more than 50% of the DDIs used by an organism, and even higher fractions of the DDIs conserved between organisms, is consistent with previous publications that reported the relatively high abundance of homodimers in PPI networks [25-27]. Ispolatov and coworkers [25] reported that both homodimers and dimers formed by paralogs are very abundant. This strengthens the notion that PPIs are more inclined to be mediated by similar elements. Our analysis brings this notion one step further, because we found homotypic interactions mediating interactions between different proteins, and not just homodimerization, both in the structural database and by our mapping. Several attempts have been made to explain the source of homodimer abundance, from improved stability, through functional suitability (for example, binding of a homodimer transcription factor to a symmetric binding site), to reduction in genome size [25-27]. However, for homotypic DDIs in different proteins these explanations do not hold, and there must be a biophysical explanation for their advantage [28]. Such an advantage may be reflected in stabilizing mutations, which will have a double effect in homotypic interactions. It is possible that such considerations played a role early in evolution, leading to self-interaction of certain single-domain proteins. Such domains may have been joined later by other domains to create multidomain proteins whose interactions are mediated through the homotypic interactions [15].

Comparisons of the DDI catalogs of the five organisms in our study confirm that the 'building blocks' of the interactions are conserved in evolution. Previously, the PPI networks themselves were compared, revealing subnetworks that were conserved in evolution [29,30]. Here we show similar findings at the domain level. Among the 1637 DDIs, which we mapped onto the PPI networks of the various organisms, 665 DDIs were mapped to PPIs of at least two organisms. The number of DDIs common to four organisms ranged from 29 to 84, where the low numbers regard DDIs common to *E. coli *and three eukaryotes. These numbers are remarkable in view of the very small overlap that was recently documented for the PPI networks of human, yeast, fly, and worm [31], when sequence similarity *per se *was used for comparison of pairs of interacting partners between species. When comparing the PPIs in the four networks only 16 common interactions were found [31], whereas we find 84 common DDIs used by these four organisms. The differences in the repertoires of shared DDIs between the *E. coli *PPI network and the networks of the three other eukaryotes, and the differences observed between the four eukaryotes are also remarkable. Although some DDIs appear to be ancient and are shared by all organisms, other DDIs have probably evolved more recently. It is possible that the source of the DDI-PPI correspondence is in interactions between pairs of single-domain proteins that occurred in various organisms at different evolutionary stages, defining the 'seeds' of the DDI catalogs. These single-domain proteins recruited additional domains, but they maintained their ability to interact through these domain pairs. Conceivably, there are such DDI seeds that evolved early in evolution and they are found in many organisms, and others that are related to more specialized processes and evolved in certain species and not others. This may explain the recurrence of the DDIs in various organisms and in various cellular contexts.

## Conclusion

By computationally mapping structurally derived pairs of interacting domains onto the PPI networks of five organisms (from *E. coli *to human), insights into the roles of these domain pairs in the interactome networks were gained. The over-representation of these interacting domain pairs in experimentally determined protein complexes corroborates the suggestion that more stable interactions in the cell are mediated by interactions between domains rather than short motifs. There are interacting domain pairs that are used repeatedly in each of the networks, and many of them are evolutionary conserved from prokaryotes to eukaryotes. In fact, there are domain pairs that are conserved in all five organisms. This latter finding is very interesting in view of recent reports that showed that the conservation of the PPIs themselves among several organisms is very low. It seems that there are interacting domain pairs that are used as the building blocks of the interactome networks and they are conserved more than the interactions themselves.

## Materials and methods

### Compiling the structural database of DDIs

We used two sources to compile a nonredundant structural database of DDIs: the 3DID [12] and iPfam [13]. These two databases (September 2005 versions) were filtered and unified as follows. For each pair of interacting domains A-B documented in each of these databases, we calculated three measures: the number of interacting amino acids in A, the number of interacting amino acids in B, and the number of amino acid-amino acid interactions between the two domains. Two domains reported in either iPfam or 3DID as interacting were included in our database if each exhibited at least three amino acids involved in the interactions and if there were at least three interactions between them. In addition, only entries with explicit amino acids were considered (entries with ambiguous names were filtered out). Also, we manually examined DDIs based on PDB structures consisting of at least two interacting molecules, in order to avoid false-positive DDIs resulting from crystal packing. At the end of the various processing steps, our database contained 2983 structurally derived DDIs.

### Compiling species-specific PPI databases

We used three public databases as sources of the PPIs: INTACT [32], DIP [19], and BIOGRID [33]. These databases consist of both literature-curated PPIs from small-scale experiments and PPIs based on high-throughput experiments. For each of the five organisms, *E. coli*, *S. cerevisiae*, *C. elegans*, *D. melanogaster*, and *H. sapiens*, we generated a nonredundant dataset of documented PPIs. Because some of the interacting proteins in human were published by their gene name, there was a concern that we may assign DDIs involving domains that actually were not included in the mature proteins because of alternative splicing. Therefore, human proteins encoded by alternatively spliced genes were excluded from the study.

### Domain assignment

We used the domain definitions of the InterPro database [34] for the assignment of domains to each of the proteins in our study. A protein may be labeled by one or several different domains. We examined what fraction of the protein sequences in our data are covered by the domains (based on the InterPro annotations), and found that on average the domains cover 91.3% of a protein's residues. In case a specific domain occurred more than once in a protein, it was assigned only once. In order to characterize the interacting domains, we used the domains' Gene Ontology (GO) annotations from the InterPro database.

### Statistical evaluations of the results

The first question we addressed was to what extent the organism's PPIs could be attributed to the structure-based DDIs. To this end, we counted the number of PPIs to which DDIs could be mapped. To evaluate the statistical significance of our findings, we generated 1000 random PPI networks for each of the five organisms, preserving the number of nodes and the degree of each node. The random networks were generated from all the proteins of an organism. For a given organism, we counted for each random network the number of PPIs to which DDIs could be mapped. The fraction of random networks where this count was equal to or exceeded the count in the actual network provided the statistical significance. Our statistical analysis guarantees that any over-representation of DDIs found in the PPI networks is not due to large families of proteins that contain these domains, because these large families were also taken into account in the generation of the random networks.

We next studied the evolutionary conservation of DDIs by comparing the DDI repertoires of the organisms. To evaluate the statistical significance, we generated 1000 random DDI networks for each of the five organisms. The random networks were generated from all the InterPro domains assigned to the organism's proteins, while preserving the number of nodes and the degree of each node in the original DDI network. We then performed 1000 comparisons between the random DDI repertoires of two or more organisms. The statistical significance of the conserved DDIs was evaluated by comparing the number of conserved DDIs between two or more organisms to the equivalent counts in the random networks, and computing the fraction of random networks in which the conserved DDI count was equal to or exceeded the actual count.

### Exclusion of interologs

Two pairs of interacting proteins in two organisms are defined as interologs if each pair-mate is an ortholog of its corresponding pair-mate in the other organism [35,36]. Such interologs might contain the same domains correspondingly and therefore may lead to a conclusion that their DDIs are conserved, whereas it is the orthology *per se *that is the basis of this finding. In order to avoid such misleading conclusions we repeated the analysis after exclusion of interologs. We determined orthlogous proteins based on three resources: The COGs database [37,38]; the Metagenes database [39], which consists of sets of genes across multiple organisms whose protein sequences are one another's best BLAST [40] hits; and the String database [41]. Interologs were omitted based on the orthology relationships and our databases of PPIs.

### Exclusion of paralogs

An additional bias in the results may be caused by paralogs. Two pairs of interacting proteins within the same organism that exhibit paralogy relationships correspondingly may lead to false conclusions about repeatedly used DDIs within an organism due to paralogy *per se*. To rule out conclusions due to such a bias, we repeated the analysis after exclusion of interacting paralogous pairs. Paralogs were determined by BLAST [40], using a strict E value threshold (10 × e^-35^), in order to avoid exclusion of two unrelated proteins that only share a domain.

## Additional data files

The following additional data are available with the online version of this paper. Additional data file 1 contains a series of supplementary figures and tables. Additional data file 2 contains lists of DDIs for *E. coli*, *S. cerevisiae*, *C. elegans*, *D. melanogaster*, and *H. sapiens*. Additional data file 3 contains lists of DDIs that are shared by two or more organisms (*E. coli*, *S. cerevisiae*, *C. elegans*. *D. melanogaster*, and *H. sapiens*).

## Supplementary Material

Additional data file 1 A series of supplementary figures and tables. Click here for file

Additional data file 2 Lists of DDIs for *E. coli*, *S. cerevisiae*, *C. elegans*, *D. melanogaster*, and *H. sapiens*; each domain pair record contains InterPro accessions, domain names, number of occurrences, and GO annotations. Click here for file

Additional data file 3 Lists of DDIs that are shared by two or more organisms (*E. coli*, *S. cerevisiae*, *C. elegans*. *D. melanogaster*, and *H. sapiens*); each domain pair record contains InterPro accessions, domain names, number of occurrences, and GO annotations. Click here for file

## Figures and Tables

**Table 1:** DDI-PPI mapping: all protein interactions

Organism^a^	*E. coli*	yeast	worm	fly	human
Number of PPIs^b^	6,934	24,699	5,315	24,703	28,800
Number of PPIs with known domains^c ^(*N*)	6,038	18,202	3,351	14,939	25,004
Number of PPIs to which DDIs could be mapped^d ^(*n*) (%; *n*/N*100) Median value of random networks (*P *value)	806 (13%) 295 (<0.001)	1,660 (9%) 363 (<0.001)	375 (11%) 142 (<0.001)	891 (6%) 276 (<0.001)	4,924 (20%) 911 (<0.001)
No. of PPIs to which DDIs could be mapped, disregarding PPI-DDI mappings due to overlap between the structural database and the PPI data (*n*)(%; *n*/N*100) Median value of random networks (*P *value)	755 (13%) 288 (<0.001)	1608 (9%) 353 (<0.001)	375 (11%) 142 (<0.001)	889 (6%) 276 (<0.001)	3,989 (16%) 856 (<0.001)

^a^Organism labeling: yeast, *S. cerevisiae*; worm, *C. elegans*; fly, *D. melanogaster*. ^b^See Figure 1a. ^c^See Figure 1c. ^d^See Figure 1e. DDI, domain-domain interaction; PPI, protein-protein interaction.

**Table 2:** DDI-PPI mapping: interactions involving only single-domain proteins

Organism^a^	*E. coli*	yeast	worm	fly	human
Number of PPIs (with known domains) (*N*)	497	2,418	335	2,068	1,633
Number of PPIs to which DDIs could be mapped (*n*) (%; *n*/N*100) Median value of random networks (*P *value)	117 (24%) 92 (0.005)	284 (12%) 79 (<0.001)	60 (18%) 38 (<0.001)	217 (10%) 55 (<0.001)	400 (25%) 135 (<0.001)

^a ^Organism labeling: yeast: *S. cerevisiae*, worm: *C. elegans*, fly: *D. melanogaster*. For the analysis of each organism, DDIs in the structural database that are based on single-domain proteins of the respective organism were excluded. DDI, domain-domain interaction; PPI, protein-protein interaction.

**Table 3:** Common and unique DDIs by pair-wise organism comparison

	*E. coli*^a^	Yeast	Worm	Fly	Human	Total
*E. coli*	**469^b ^(62%)^c^**	211 (28%)	79 (10%)	64 (8%)	178 (23%)	759
yeast	211 (36%)	**106 (18%)**	163 (28%)	164 (28%)	352 (61%)	579
worm	79 (31%)	163 (65%)	**24 (10%)**	118 (47%)	193 (77%)	251
fly	64 (24%)	164 (60%)	118 (43%)	**8 (3%)**	239 (88%)	272
human	178 (20%)	352 (39%)	193 (22%)	239 (27%)	**365 (41%)**	897

^a^Organism labeling: yeast, *S. cerevisiae*; worm: *C. elegans*; fly, *D. melanogaster*.

^b^Values in bold represent the DDIs unique to this organism. ^c^The percentage is calculated out of the total number of the organism's DDIs (right most column). The percentages in each line do not add up to 100% because there are DDIs that are shared by more than two organisms and they are counted more than once. DDI, domain-domain interaction.

**Table 4:** DDI-PPI mapping based on both single domain PPIs and the structural DDIs

Organism^a^	*E. coli*	yeast	worm	fly	human
Number of PPIs	6,934	24,699	5,315	24,703	28,800
Number of PPIs with known domains (*N*)	6,038	18,202	3,351	14,939	25,004
Number of PPIs to which DDIs could be mapped (*n*) (%; *n*/N*100)	1,234 (20%)	5,048 (28%)	860 (26%)	3,983 (27%)	7,991 (32%)

^a ^Organism labeling: yeast: *S. cerevisiae*, worm: C. elegans, fly: *D. melanogaster*. DDI, domain-domain interaction; PPI, protein-protein interaction.
