# Supplementary material for: Evolutionary conservation of domain-domain interactions
Source: Genome Biol. 2006 Dec 21;7(12):R125. doi: 10.1186/gb-2006-7-12-r125 (PMC1794438; doi:10.1186/gb-2006-7-12-r125)
Supplement: Additional data file 1 — A series of supplementary figures and tables. [file gb-2006-7-12-r125-S1.pdf]

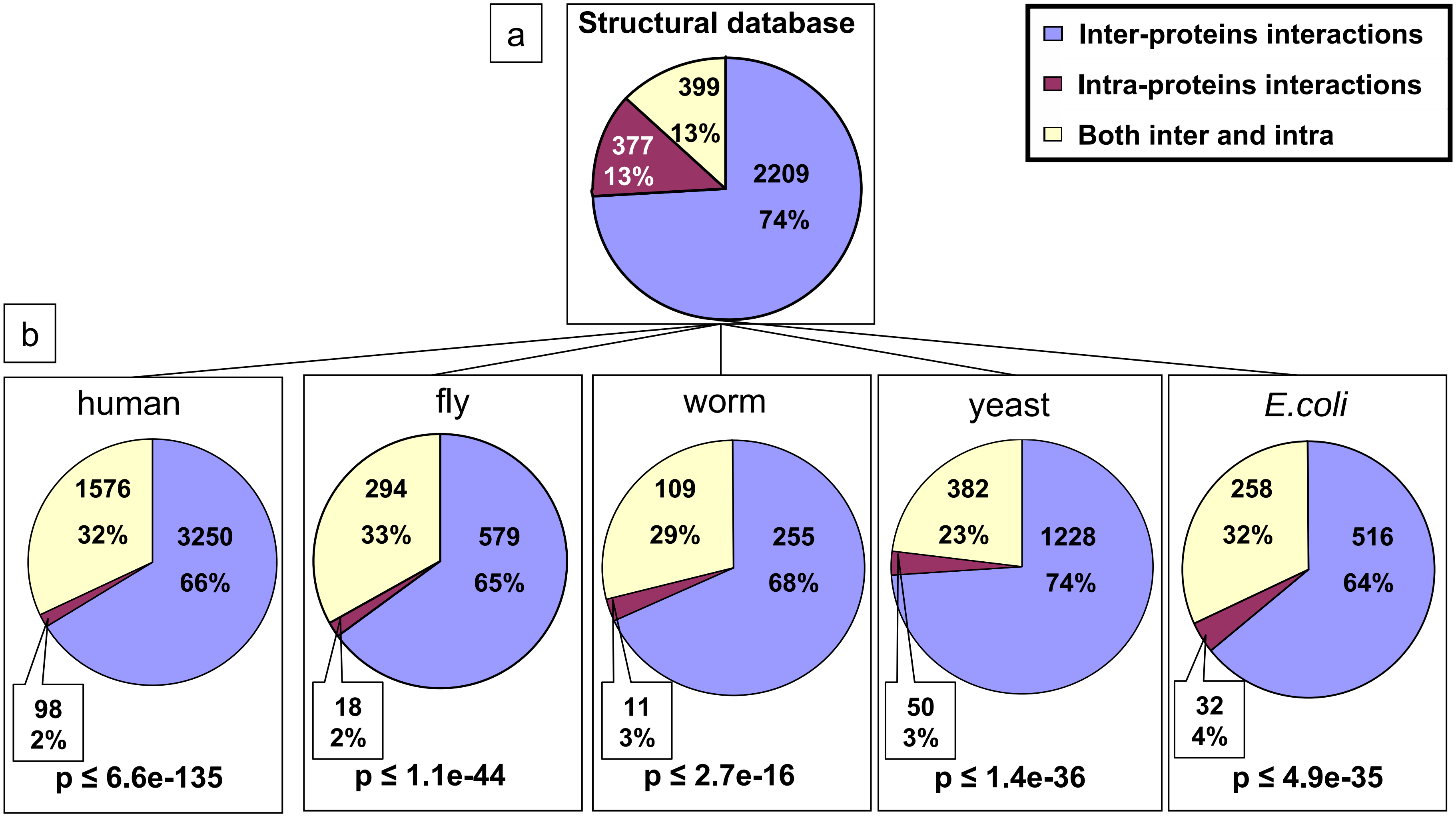

Figure 1

Types of structurally-derived interacting domain-pairs used in cellular protein-protein interaction networks

The structurally-derived DDIs were based on intra-protein interactions, inter-protein interactions, or both.

The pie-charts show that the distribution of these three types in each of the networks of the studied organisms differs significantly from the distribution in the structural database (p-values were determined by a $\chi 2$ test). A very small fraction of the DDI mappings in the cellular networks are based on intra-protein DDIs. Most mapping of DDIs to PPIs in all organisms are based on inter-protein interactions and to a lesser extent on DDIs that were found in both inter- and intra-protein interactions.

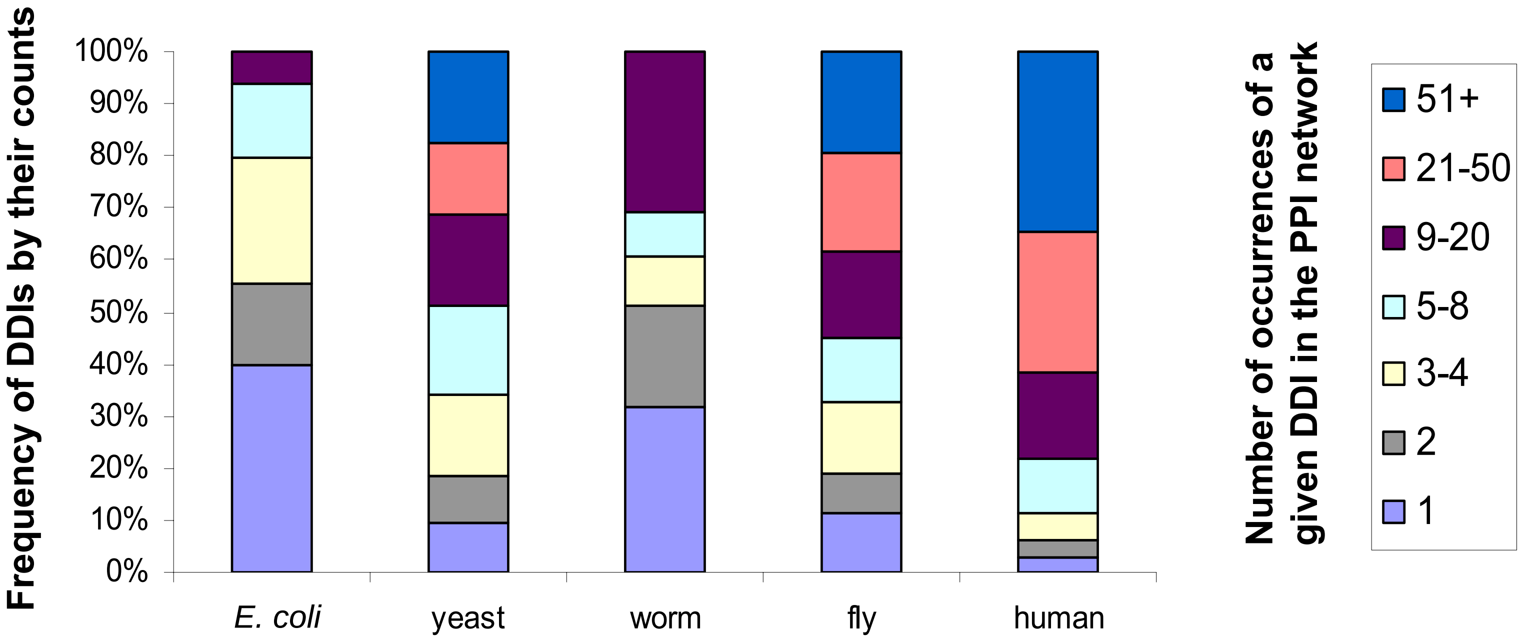

Figure 2

Repeated use of interacting domain-pairs in protein-protein interaction networks without paralogs

For each organism, the number of occurrences of each DDI in the PPI network without the paralogs was counted. The histogram shows the frequency of PPIs that were attributed to DDIs used only once, twice, etc. The frequency is computed out of all the PPI-DDI mappings.

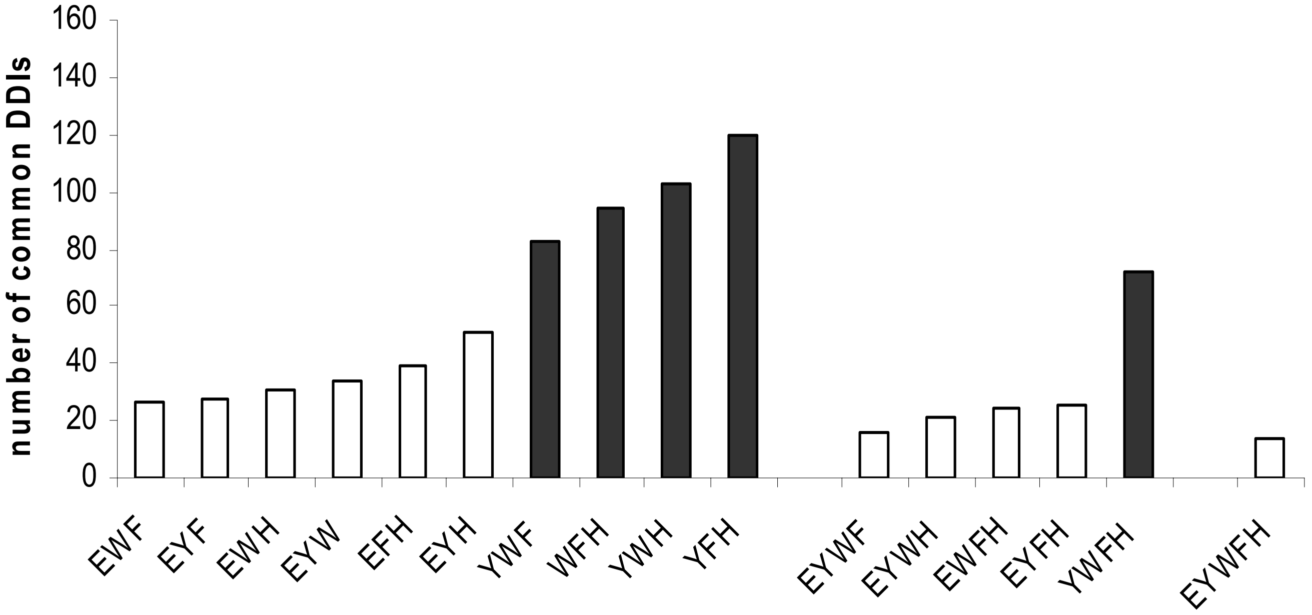

Figure 3

Interacting domain-pairs common to three, four and five organisms without interologs

The histogram shows the number of DDIs shared by three, four and five organisms. White bars represent DDIs that are used also in *E. coli* and black bars represent DDIs common only to the eukaryotes in our study. All of the results were statistically significant ($p<0.001$).

E: *E. coli*. Y: yeast (*S. cerevisae*). W:worm (*C. elegans*). F:fly (*D. melanogaster*). H:human

Table 1

Repeatedly used DDIs in the networks, based on non-paralogous PPIs

The organism[1]	*E.coli*	Yeast	Worm	Fly	Human
Total DDIs	759	579	251	272	897
Repeating DDIs (found more than once)[a]	371	341	109	137	598
Repeating DDIs that are not based exclusively on paralogous proteins[b]	81	244	61	120	406
Ratio[(b/a)]	0.22	0.72	0.56	0.88	0.68

[1] Organism labeling: Yeast: *S. cerevisiae*, Worm: *C. elegans*, Fly: *D. melanogaster*

Table 2

Counts of conserved DDIs, based on the studied PPI networks, with and without interologs

E.coli	yeast[1]	worm[1]	fly[1]	Human	Number of common DDIs (p-value)	Number of common DDIs without interologs[2] (p-value)
+	+				211 (<0.001)	87 (<0.001)
+		+			79 (<0.001)	73 (<0.001)
+			+		64 (<0.001)	61 (<0.001)
+				+	178 (<0.001)	120 (<0.001)
	+	+			163 (<0.001)	147 (<0.001)
	+		+		164 (<0.001)	154 (<0.001)
	+			+	352 (<0.001)	280 (<0.001)
		+	+		118 (<0.001)	115 (<0.001)
		+		+	193 (<0.001)	178 (<0.001)
			+	+	239 (<0.001)	219 (<0.001)
+	+	+			67 (<0.001)	34 (<0.001)
+	+		+		51 (<0.001)	28 (<0.001)
+	+			+	108 (<0.001)	51 (<0.001)
+		+	+		32 (<0.001)	26 (<0.001)
+		+		+	57 (<0.001)	31 (<0.001)
+			+	+	54 (<0.001)	39 (<0.001)
	+	+	+		89 (<0.001)	83 (<0.001)
	+	+		+	136 (<0.001)	103 (<0.001)
	+		+	+	147 (<0.001)	120 (<0.001)
		+	+	+	110 (<0.001)	94 (<0.001)
+	+	+	+		29 (<0.001)	16 (0.002)
+	+	+		+	49 (<0.001)	21 (<0.001)
+	+		+	+	46 (<0.001)	25 (<0.001)
+		+	+	+	30 (<0.001)	24 (<0.001)
	+	+	+	+	84 (<0.001)	72 (<0.001)
+	+	+	+	+	27 (<0.001)	14 (0.001)

[1]Organism labeling: yeast: *S. cerevisiae*, worm: *C. elegans*, fly: *D. melanogaster*.

[2]The relatively large decrease in the number of DDIs common to *E.Coli* and yeast after excluding the interologs, is mainly due to the relatively high number of *E.coli*-yeast orthologs in the databases we used (see methods).
